# Association between temperature exposure and cognition: a cross-sectional analysis of 20,687 aging adults in the United States

**DOI:** 10.1186/s12889-021-11533-x

**Published:** 2021-07-29

**Authors:** Anam M. Khan, Jessica M. Finlay, Philippa Clarke, Ketlyne Sol, Robert Melendez, Suzanne Judd, Carina J. Gronlund

**Affiliations:** 1 Department of Epidemiology, School of Public Health, University of Michigan, 1415 Washington Heights, Ann Arbor, MI 48109-2029 USA; 2 Social Environment and Health, Institute for Social Research, University of Michigan, 426 Thompson Street, Ann Arbor, MI 48104 USA; 3 Department of Biostatistics, University of Alabama at Birmingham, Ryals Public Health Building (RPHB), 1665 University Boulevard, Birmingham, AL 35233 USA

**Keywords:** Temperature, Extreme heat, Extreme cold, Cognitive performance, Climate and context, Aging, Older adults, Dlnm, REGARDS

## Abstract

**Background:**

Older adults are particularly vulnerable to the adverse health effects of extreme temperature-related events. A growing body of literature highlights the importance of the natural environment, including air pollution and sunlight, on cognitive health. However, the relationship between exposure to outdoor temperatures and cognitive functioning, and whether there exists any differences across climate region, remains largely unexplored. We address this gap by examining the temperature-cognition association, and whether there exists any variation across climate regions in a national cohort of aging adults.

**Methods:**

In this cross-sectional study, we obtained data on temperature exposure based on geocoded residential location of participants in the REasons for Geographic And Racial Differences in Stroke (REGARDS) study. For each participant, this information was linked to their cognitive scores from Word List Learning and Recall tests to assess cognitive functioning. We used distributed lag non-linear models (dlnm) to model temperature effects over 2 days. Multivariable linear regression was used to compute temperature-cognitive functioning associations, adjusted for important covariates. Region-specific (“Dry”, “Mediterranean/oceanic”, “Tropical” and “Continental”) associations were examined by including an interaction term between climate region and temperature.

**Results:**

Amongst 20,687 individuals (mean age = 67.8; standard deviation = 9.2), exposure to region-specific extreme cold temperatures in the “dry” region (e.g., Arizona) over 2 days was associated with lower cognitive scores (Mean Difference [MD]: -0.76, 95% Confidence Interval [CI]: − 1.45, − 0.07). Associations remained significant for cumulative effects of temperature over 2 days. Extremely cold exposure in the “Mediterranean/oceanic” region (e.g., California) over 2 days was also associated with significantly lower cognitive performance (MD: -0.25, 95% CI: − 0.47, − 0.04). No significant associations were observed for exposure to hot temperatures. Cognitive performance was slightly higher in late summer and fall compared to early summer.

**Conclusion:**

We noted adverse cognitive associations with cold temperatures in traditionally warmer regions of the country and improved cognition in summer and early fall seasons. While we did not observe very large significant associations, this study deepens understanding of the impact of climate change on the cognitive health of aging adults and can inform clinical care and public health preparedness plans.

**Supplementary Information:**

The online version contains supplementary material available at 10.1186/s12889-021-11533-x.

## Background

The increasing frequency and intensity of extreme weather events pose substantial human and financial costs [1]. Studies note that extreme weather events are associated with increased mortality, hospitalization rates and emergency department visits, particularly among older adults [2–6]. The health effects of extreme ambient temperatures, such as those experienced during the European heat wave of 2003, are consequential [7]. The burden on human health is only expected to grow in magnitude in coming years with climate change [7]. Furthermore, the effects may vary based on whether regions have traditionally warm or cold climates, although further study in this area is required [8].

Weather may adversely impact health outcomes including cognitive functioning. Cognitive decline, dementia and Alzheimer’s disease are pressing medical and population health issues given their substantial morbidity, cost and caregiving burden [9]. Previous studies have largely focused on identifying individual-level risk factors (e.g., age and comorbid conditions), yet recent evidence suggests that the surrounding environment may also impact cognitive performance [10, 11].

In evolving socio-ecological models of cognitive health, the role of the natural environment - particularly temperature - is not well characterized. Environmental exposures that may affect cognitive function include air pollution, sunlight, and seasonality; although further study of the seasonal effects, fully accounting for temperature, is required [12–15]. Outside of experimental studies, literature examining heat and cognition demonstrates that participants exposed to hot temperatures have slower reaction times and commit more errors on cognitive tests [16, 17]. These observational studies are usually small in size and/or conducted in younger or selective populations. Furthermore, these studies are geographically restricted in scope (e.g., conducted in a single city, area, or state). Thus, little is known about temperature-cognition associations across different climate regions. Cold temperatures may also be associated with impaired memory, as studied in laboratory settings or in selective populations. The results are mixed, with both increases and decreases in accuracy and efficiency on cognitive tests assessing vigilance, reasoning, and memory [17, 18]. Despite the limited evidence amongst older adults, it is plausible that exposure to extreme temperatures can impact cognitive health. Their central nervous system is more sensitive to internal temperatures; higher internal temperatures might lead to memory impairments and other morbid conditions [7, 19].

Older adults may be particularly vulnerable to the adverse effects of extreme temperatures. They are more sensitive to changes in the environment due to factors such as age-related changes in thermoregulation, internal body temperature, medication use and a higher burden of comorbid conditions [20–24]. The cumulative effect of these factors often makes organ systems less able to tolerate stress induced by extreme temperatures [7]. Moreover, higher rates of social isolation and more limited financial resources can restrict older adults’ abilities to engage in adaptive behaviors in response to adverse weather events [25]. This underscores the importance of investigating the role of temperature on morbidity as people age.

Therefore, in this cross-sectional study, we used data from the REasons for Geographic And Racial Differences in Stroke (REGARDS) study to examine the association between short-term temperature exposure and cognition in a large national sample of aging adults in the United States. We hypothesized that both cold and hot temperatures would be associated with decreased cognitive performance. Given the geographic diversity of REGARDS participants, we further examined potential differences by climate region. This study contributes new insights regarding temperature and cognitive health in aging adults that can inform public health, policy and clinical care.

## Methods

### Data

We performed a population-based cross-sectional study using data from the REGARDS study. REGARDS is an ongoing national study in the United States (US). Approximately 30,000 non-Hispanic Black and White men and women who were at least 45 years at baseline were recruited between 2003 and 2007 (mean age at baseline was > 65 years) [26, 27]. The study aims to identify factors that contribute to excess stroke mortality among Black Americans, particularly those living in the Stroke Belt, with oversampling in these populations [26]. At baseline, investigators collected detailed socio-demographic, lifestyle, medical and cognitive information. Various cognitive tests are administered to participants on an annual or bi-annual basis, and residential addresses are tracked during follow-up [26]. REGARDS study investigators obtained written informed consent from all participants. The study procedures are reviewed and approved by the Institutional Review Board at the University of Alabama at Birmingham (IRB-020925004).

### Cognitive assessments

Together, the Word List Learning (WLL) and Word List Delayed Recall (WLD) tests measure cognitive performance, specifically, episodic memory, which is an important marker of cognitive health. We chose measures of episodic memory in the current study because decline in episodic memory is an early predictor of dementia development [28]. With Azheimer’s disease being the most common type of dementia and a leading cause of death in older age, episodic memory may be one way of examining the relationship between temperature, cognitive health and other later life health outcomes [9]. A major advance of this study is the use of a sub-clinical health outcome and our ability to investigate temperature associations with cognition before the individual suffers an event severe enough to warrant seeking medical care. The WLL and WLD were first administered in REGARDS in 2006. The WLL involved verbally reciting to participants a list of ten words for immediate recall in three, consecutive trials (score range: 0–30), with the WLD involving a delay before recall in a single trial (score range: 0–10). Higher scores indicate better cognitive performance (i.e., more words remembered). We used the participant’s first available WLL and WLD measures to create a composite index of these tests by converting each to a z-score based on the mean and standard deviation of that test, and taking the average of the z-score for the two tests.

### Temperature assessment

We examined temperature exposure on the day of testing and up to 2 days prior by linking to county-level temperature data from NOAA weather stations to each participant [29]. Mean temperature on a given day was computed by averaging the maximum and minimum temperatures reported for weather stations in a participant’s county over a 24-h period. These values were then averaged across all the weather stations in a county.

### Covariates

A variable for the year of testing was included to address time trends in test administration and climate. We selected confounders and covariates a priori. These were variables thought to influence regional variation in temperature exposure, and cognition. Age (in years, at the time of the test), education level (less than high school, high school diploma, some college education, college degree or higher), race (Black or White), and sex (Male or Female) were included. Lifestyle factors such as physical activity, and comorbid conditions were conceptualized as mediators in the research question of interest and were thus not included in the regression model.

Participants in the REGARDS cohort were administered cognitive tests at different times of the year (e.g., summer or winter). To account for this, in the main analysis examining the association between temperature exposure and cognition, we considered seasonality as the numbered day in the year (1–364) when the test was administered, modeled as a sinusoidal function. In a separate analysis aimed at examining the season-cognition association (adjusted for temperature), we ran a regression model with season modeled using natural cubic splines of day-of-year with 4 degrees of freedom (*df*). This analysis allows us to capture the time-varying relationship of temperature, with control for season. Seasonal effects may also include light and pollen exposure, and some studies have noted light and pollen exposure to be associated with cognitive processes or performance [30, 31]. Individuals were assigned to Köppen climate regions based on their geocoded locations. This is a commonly used classification system in climate studies. It groups climates into five broad types based on seasonal precipitation and temperature that can be further subdivided [32]. Due to limited sample sizes in some of the sub-divisions, we used the highest level of classification and grouped Köppen climates into one of four categories (regions named: “dry”, “continental”, “tropics”, and “Mediterranean/oceanic”).

### Analytical sample

Of 30,239 participants in the REGARDS study at the time of this analysis, we excluded participants missing a WLL or WLD cognitive assessment (*N* = 6302). Individuals who had a stroke prior to their first assessment or self-reported a stroke at time of study commencement (*N* = 1580) were not included owing to the challenges of disentangling the cognitive effects of stroke and recovery from the effects of the exposure [33]. Furthermore, we excluded participants missing residential location information or valid data from weather stations on the day of their first WLL and WLD tests (*N* = 1661). We excluded a further 9 participants who could not be assigned to a Köppen climate region or were missing socio-demographic information. The final analytic sample was 20,687.

### Statistical analyses

In a cross-sectional study design, we computed descriptive statistics in the overall sample and by region. We calculated the frequency and percentage for binary and categorical variables and the mean and standard deviation for continuous variables.

We modeled temperature associations using natural cubic splines with 2 *df*. Natural cubic splines afford greater flexibility at the endpoints of temperature distribution, where non-linearity might occur, and are commonly used in studies of temperature and health [34]. We modeled temperature using up to 7 *df*, but a consistent functional form was observed when we specified 3–7 *df* (See Additional File 1, Fig. 1). In addition, the Bayesian Information Criterion (BIC) and Akaike Information Criterion (AIC) both increased incrementally with the inclusion of additional *df*. Therefore, a model with 2 *df* was selected for model parsimony.

**Fig. 1:**
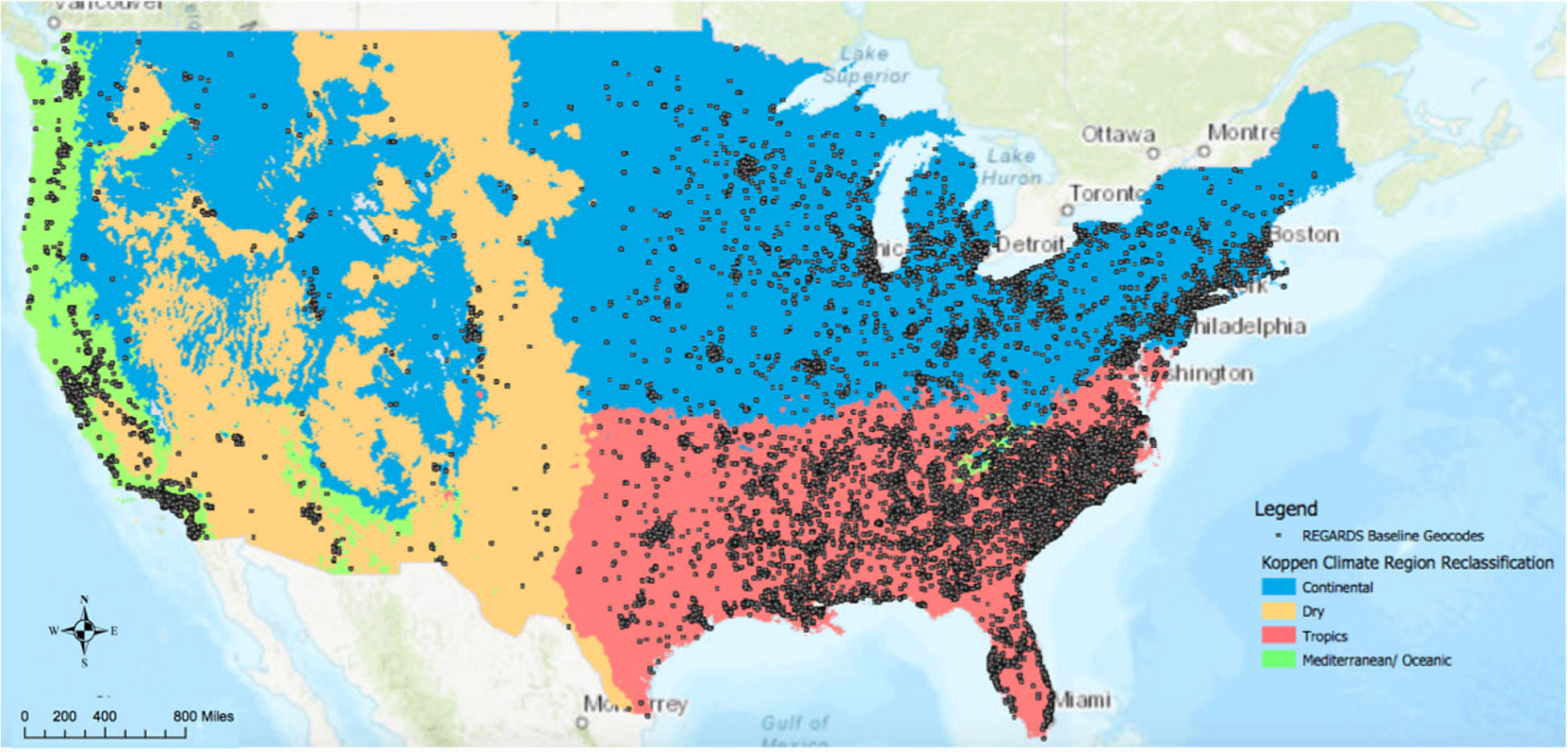
Classification of Köppen climate regions. Grey dots represent the location of a participant in the REasons for Geographic and Racial Differences in Stroke (REGARDS) study at the time of the baseline interview. Sources: Idaho State Climate Services (1999). Köppen Climate Classification for the Conterminous United States, ESRI GRID File. Idaho Geospatial Data Clearninghouse [producer]. DATA.GOV [distributor], 2017. Web. July 2019. https://catalog.data.gov/dataset/koppen-climate-classification-for-the-conterminous-united-states63aa7

Using previously described methods in the literature, we also considered the impact that temperature exposure on the days prior to the date of testing, termed temperature lags, might have on cognitive performance [35, 36]. This is a natural choice to appropriately model the temperature-cognition association, informed by findings in temperature-mortality studies that cold effects are often spread over a week or more after a cold day, whereas the heat effect is more immediate [35]. We modeled lag and temperature effects simultaneously using a crossbasis function using the dlnm package in R, a widely used method for characterizing the substantially nonlinear and lagged effects of acute temperature exposure on health [35, 36]. Each participant’s vector of lagged temperature exposures were recast as a vector of values representing natural cubic splines in the temperature and lag dimensions, with 2 *df* in both the temperature and lag dimensions to allow nonlinear effects. This allows for temperature effects to be stronger at both high and low temperature values, and to allow lagged effects to be accounted for but to differ in magnitude from same-day (lag day 0) effects [35]. A feature of these distributed lag models is that “cumulative lag effects” can either be interpreted as the effect of temperatures on the days leading up to a single day of cognitive testing or as the effect of a single day of temperature on cognition on the following days. Hence “cumulative lag day 0-1 effects” refers to the effects of temperature on cognition on the day of testing *plus any delayed effects* on cognition on the day following testing [35]. We performed an exploratory analysis accounting for delayed effects of temperatures up to 7 days after date of testing, but no significant associations on cognition were observed beyond 2 days (See Additional File 1, Fig. 2). Therefore, we focused on cumulative association of exposure to temperature over 3 days (lag days 0–2). We examined the following lag days for outdoor temperature: day of testing (lag 0), day of testing and the day following (lag days 0–1), and day of testing and up to 2 days following (lag days 0–2). Computing the cumulative association enabled us to examine the *increased risk* over the 3 days due to 1 day’s temperature exposure. In the final model, we assigned 2 *df* in both the temperature and lag dimensions.

**Fig. 2:**
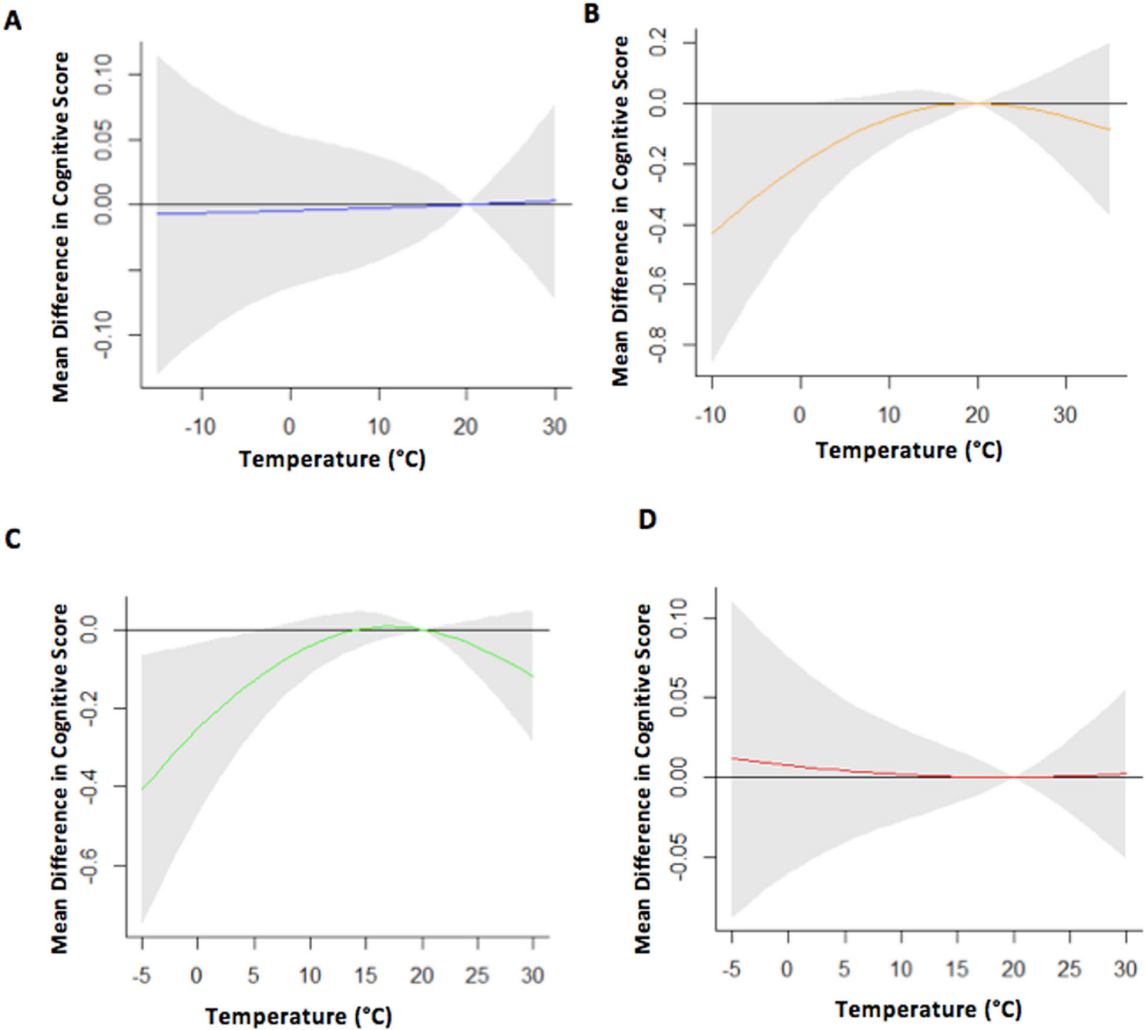
Region specific association between temperature (°C) and cognition. The association was examined across the continental (**A**), dry (**B**), Mediterranean/oceanic (**C**), and Tropics (**D**) Köppen climate regions, accounting for the cumulative effect of temperature on the day of testing and the 2 days prior. Reference for temperature was 20 °C. Models adjusted for age at time of cognitive assessment, year of test, season, education status at baseline, sex and race. Grey shaded area represents the 95% confidence around the effect estimate

Multivariable linear regression modeled the overall association between temperature (represented using the temperature crossbasis) and cognitive performance, adjusted for covariates. We centered the splines at room temperature (20 **°**C). To examine potential variation by region, a subsequent model included interaction terms between the temperature crossbasis and Köppen climate region. To help visualize the region-specific temperature-cognition relationship, we estimated and plotted the mean difference (MD) and 95% Confidence Intervals (CI) for the relevant associations across the temperature distribution for lag day 0, lag days 0–1 and lag days 0–2. Due to the use of natural cubic splines to model temperature, effect estimates from the regression model cannot be interpreted directly. Instead, specific values across the temperature distribution have to be selected and reported. Therefore, for each climate region, we estimated the cumulative effects of region-specific 1st (extreme cold), 10th (cold), 90th (hot) and 99th (extreme heat) percentile temperatures for the day of testing and effects up to 2 days. In a separate model, we estimated the independent association of season (modeled using natural cubic splines of day-of-year with 4 *df)* and cognition for specific days of the year corresponding to different seasons. The spline centered on the day number of the year corresponding to May 15th (early summer).

Statistical tests were 2-sided and we set *P* < 0.05 as the level for statistical significance. SAS version 9.4 (SAS Institute, Cary, North Carolina) was used for data management and R Studio to perform the analysis.

## Results

The study population had a mean age of 67.8 years (standard deviation (SD): 9.2 years) and a majority of participants were White (Table 1). The population was highly educated with around half having at least some College-level education (Table 1). The mean composite cognitive score and daily mean temperature in the overall cohort and across regions are noted in Table 1. Average daily mean temperature countrywide on the day of testing was 15.6 °C (SD: 9.4 °C). Across the regions, this ranged from 10.6 °C in the “continental” climate region to 17.5 °C in the tropics climate region (Table 1).

**Table 1:** Characteristics of the Study Cohort within the REasons for Geographic and Racial Differences in Stroke (REGARDS) Study by Climate Region

Characteristic	Dry	Continental	Tropics	Mediterranean/oceanic	Overall
	N (%), unless otherwise specified				
Participants (N)	485	5026	13,494	1682	20,687
Age, mean (SD), years^a^	68.9 (9.5)	68.3 (9.3)	67.5 (9.1)	68.3 (9.8)	67.8 (9.2)
Sex, female	247 (50.9)	2711 (53.9)	7794 (57.8)	1024 (60.9)	11,776 (56.9)
Race, Black	43 (8.9)	2111 (42.0)	5062 (37.5)	759 (45.1)	7975 (38.6)
Education					
Less than high school	13 (2.7)	461 (9.2)	1478 (11.0)	58 (3.5)	2010 (9.7)
High school graduate	92 (19.0)	1355 (27.0)	3490 (25.9)	231 (13.7)	5168 (25.0)
Some College	140 (28.9)	1347 (26.8)	3569 (26.5)	515 (30.6)	5571 (26.9)
College graduate and above	240 (49.5)	1863 (37.1)	4957 (36.7)	878 (52.2)	7938 (38.4)
Temperature on day of test in °C, mean (SD)^b^	15.2 (9.1)	10.6 (10.8)	17.5 (8.4)	15.5 (6.2)	15.6 (9.4)
Composite cognitive score, mean^c^	0.1 (0.9)	−0.05 (0.9)	−0.09 (1.0)	0.05 (0.9)	− 0.06 (1.0)

*Abbreviations*: *N* number, *SD* standard deviation

Climate regions were created based on the climate groups within Köppen climate region classification system. Individuals residing in Humid Subtropical climates were combined with those living in the Tropics to form one group and the Mediterranean and Oceanic climates were grouped to form the Mediterranean/oceanic climate region, to ensure adequate sample size

^a^ Age at the time of the first available Word List Learning (WLL) and Word List Delayed (WLD) recall test score over the course of the study from 2006 to 2016

^b^ Temperature was computed as the mean of minimum and maximum temperature reading on the day of the cognitive assessment for each weather station, and averaged across all the National Oceanic and Atmospheric Administration (NOAA) weather stations in the county of the participant

^**c**^ Score was generated by averaging the z-scores for the WLL and WLL-D in the entire cohort

Participant characteristics (age, race, sex and education) were self-reported at the time of the baseline interview between 2003 and 2007

Figure 1 indicates the distribution of participants across the Köppen climate categories. The dry region encompassed areas in the Southwest. The continental, tropics and Mediterranean/oceanic regions were largely found in the Northeast, Southeast, and near the Pacific coast, respectively. Most of the cohort (~ 65%) was located in the tropics region. This is owing to oversampling in the Stroke Belt of the country, a geographic area that comprises a large portion of the tropics climate region (Fig. 1).

We noted region-specific differences in the temperature-cognition association, although no significant temperature-cognition associations were observed across any temperature values in the pooled analyses (See Additional File 1, Fig. 3).

**Fig. 3:**
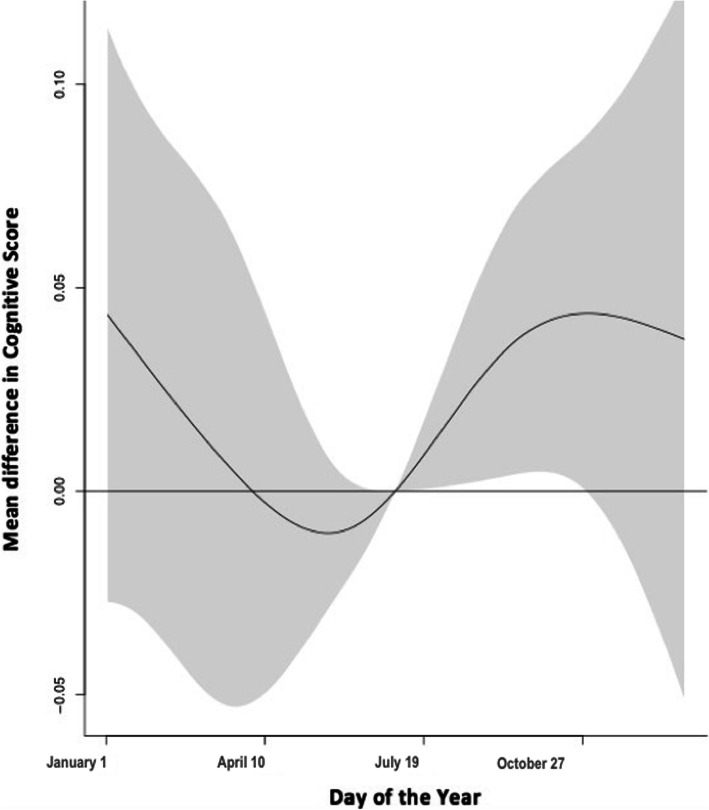
Season effect on cognition. Mean difference in composite cognitive score for day of the year versus reference value of May 15th. Models adjusted for age at time of cognitive assessment, year of test, temperature, education status at baseline, sex, race and climate region. Grey shaded area represents the 95% confidence around the effect estimate. Season was modeled as day number of the year

When examining the cumulative effect of temperature over 3 days, the observed functional forms for the dry and Mediterranean/oceanic regions were concave in shape. We observed an inverse association between temperature and memory in these regions for colder temperatures whilst the association was null for warmer temperatures (Fig. 2B and C). The associations in the other regions were null across all temperatures examined (Fig. 2A and D).

Significant associations between temperature and cognition were evident in the dry and Mediterranean/oceanic climate regions. For cumulative associations of temperature across 3 days, exposure to region-specific extreme cold temperatures (0 °C) in the Mediterranean/oceanic climate region was associated with a 0.25 point lower cognitive score (MD: -0.25, 95% CI: − 0.47, − 0.04) (Table 2). Associations for cold temperature were not statistically significant when temperature on the day of testing and/or the day prior were considered in this region (Table 2). Among those residing in the dry climate region, significantly lower cognitive scores were observed for exposure to region-specific cold temperatures (4 °C) on day of testing (MD: -0.41, 95% CI: − 0.75, − 0.08) (Table 2). Observed associations were more pronounced when considering cold effects of temperature on the day of the test and the day before (MD: -0.46, 95% CI: − 0.80, − 0.11). Extreme cold (− 8 °C), considering temperature on the day of testing and the day prior, was associated with scores that were 0.76 points lower (MD: -0.76, 95% CI: − 1.45, − 0.07). The cumulative effect of temperature on the day of testing and the 2 days prior waned but remained statistically significant (Table 2). We observed no significant findings for the other regions or in examining exposure to extreme hot temperatures, though confidence intervals were large.

**Table 2:** Mean difference in composite cognitive score for exposure to region-specific extreme cold (1st percentile), cold (10th percentile), hot (90th percentile), and extreme hot (99th percentile) temperatures

	Dry		Continental		Tropics		Mediterranean/oceanic	
1st percentile (Extreme cold)^**a**^	*MD*	*95% CI*	*MD*	*95% CI*	*MD*	*95% CI*	*MD*	*95% CI*
Day of testing	−0.63	−1.30, 0.03	− 0.01	− 0.20, 0.18	−0.04	− 0.13, 0.05	0.00	− 0.33, 0.35
Lag day 0–1	**− 0.76**	**−1.45, − 0.07**	−0.01	− 0.21, 0.19	−0.03	− 0.13, 0.07	−0.08	− 0.44, 0.28
Lag day 0–2	**− 0.38**	**−0.76, − 0.01**	−0.01	− 0.12, 0.11	0.01	− 0.07, 0.09	**−0.25**	**− 0.47, − 0.04**
**10th percentile (cold)**^**b**^								
Day of testing	**−0.41**	**− 0.75, − 0.08**	−0.03	− 0.15, 0.10	−0.02	− 0.07, 0.04	0.04	− 0.12, 0.20
Lag day 0–1	**− 0.46**	**−0.80, − 0.11**	−0.03	− 0.16, 0.09	−0.02	− 0.07, 0.04	0.02	− 0.15, 0.18
Lag day 0–2	− 0.13	−0.27, 0.01	− 0.01	−0.07, 0.06	0.00	−0.05, 0.05	− 0.07	−0.16, 0.02
**90th percentile (Hot)**^**c**^								
Day of testing	0.23	−0.03, 0.49	0.02	−0.03, 0.10	−0.01	− 0.06, 0.05	−0.04	− 0.12, 0.05
Lag day 0–1	0.22	−0.05, 0.49	0.02	−0.04, 0.11	− 0.01	−0.06, 0.05	− 0.05	−0.14, 0.04
Lag day 0–2	−0.02	− 0.14, 0.09	0.00	− 0.05, 0.05	0.00	− 0.04, 0.04	−0.03	− 0.09, 0.02
**99th percentile (Extreme hot)**^**d**^								
Day of testing	0.44	−0.09, 0.97	0.04	−0.03, 0.13	−0.01	− 0.08, 0.06	−0.08	− 0.27, 0.10
Lag day 0–1	0.42	−0.14, 0.97	0.05	−0.04, 0.14	− 0.01	−0.09, 0.07	− 0.11	−0.31, 0.09
Lag day 0–2	−0.07	− 0.31, 0.17	0.00	− 0.05, 0.07	0.00	− 0.05, 0.06	−0.09	− 0.21, 0.04

*Abbreviation*s: *CI* confidence interval, *MD* mean difference

Lag day 0–1 represents the cumulative effect of temperature on day of testing plus the effect of temperature from 1 day prior. Lag 0–2 is the cumulative effect of temperature on the day of testing plus 1 and 2 days prior. These can also be interpreted as the effect of a single day of temperature on cognition in the following 0–1 days or the following 0–2 days

Reference value was 20 °C

^a^ 1st percentile values were − 8 °C, − 14 °C, 0 °C and − 2 °C for dry, continental, Mediterranean/oceanic and tropics, respectively.

^b^ 10th percentile values were 4 °C, − 4 °C, 8 °C and 5 °C for dry, continental, Mediterranean/oceanic and tropics, respectively.

^c^ 90th percentile values were 27 °C, 24 °C, 24 °C and 28 °C for dry, continental, Mediterranean/oceanic and tropics, respectively.

^d^ 99th percentile values were 33 °C, 29 °C, 28 °C and 31 °C for dry, continental, Mediterranean/oceanic and tropics, respectively.

Models adjusted for age at time of cognitive assessment, year of test, season, education status at baseline, sex and race

Bolded estimates represent statistically significant findings at the *p* < 0.05 level

Season was associated with cognitive functioning, independent of temperature, though confidence intervals were large. Cognitive performance was significantly higher in late summer (MD August 31st vs May 15th: 0.04, 95% CI: 0.01, 0.08) and fall (MD October 31st vs May 15th: 0.05, 95% CI: 0.02, 0.09) compared to early summer (Fig. 3). Compared to early summer, we observed no significant associations during spring or winter.

## Discussion

Within this large, national sample of aging adults, the relationship between outdoor temperature and cognitive performance varied by climate region. We found significant adverse associations between cold temperatures and cognitive performance amongst individuals residing in the Mediterranean/oceanic and dry climate regions. Despite the large confidence intervals, cognitive function was higher in the late summer and fall when compared to mid-summer, although this association was small. The findings suggest that cognitive performance on tests of episodic memory may be sensitive to temperature but this varies by region of residence, as well as season.

Few observational studies have examined the associations between cold temperature and cognition. To our knowledge, the only previous population-based study to examine this was among male military veterans in Massachusetts, which found a ‘U’ shaped effect of spatiotemporally calculated temperature and performance on the Mini-Mental State Exam (MMSE) [17]. The effects of low MMSE were most pronounced at the extreme cold and hot ends of the distribution [17]. Impairment of short-term memory has also been reported in lab settings and amongst divers and skiers, even with brief cold exposures [18, 37–39]. Literature in this area is inconsistent, with mixed findings for logical reasoning, planning, accuracy, response times, and efficiency [39, 40].

Comparisons across studies are challenging owing to the variety of tests used on different domains of cognitive functioning. For example, tasks involving working memory and timed tasks may be more affected by cold temperature [39]. Differences in the populations examined may also explain these findings. Previous studies largely focused on groups more accustomed to cold conditions (e.g., alpine skiers) [38]. Athletes regularly exposed to colder temperatures may be less susceptible to adverse cognitive effects of cold weather due to physiological habituation responses [41]. Additionally, older adults may have different responses to temperatures as compared to younger adults given physiologic differences such as decreased ability to maintain core temperature and reduced thermal perception [7].

Our findings of adverse cognitive associations with cold temperatures were region-specific, occurring among those living in the generally milder Mediterranean/oceanic and dry climate regions of the US. To our knowledge, the temperature-cognition association has not previously been studied across regions despite previous evidence that temperature effects of other health outcomes show regional differences [6, 8, 42]. In the present study, those living along the Pacific coast and in the Southwest, which traditionally experience mild average temperatures (e.g., California: 18 °C, annual average range: 13–22 °C; Arizona: 23 °C, annual average range: 17–30 °C), were most affected by cold temperatures [43, 44]. While we were unable to assess the potential adaptive mechanisms or housing conditions of the aging adults in the REGARDS cohort, state data reports higher numbers of older homes, lower heating use, and fewer households with double/triple window panes (which are better at preserving heat within the house) in these areas compared to the national average [45, 46]. As a result, older residents may be less accustomed and equipped to counter the effects of colder temperatures in these regions, and thus may be more susceptible to the adverse effects by the time cognitive tests were administered. This may be especially true if older adults tend to spend more time in their homes and may have less access to local resources [47, 48]. Findings highlight the potential role that personal and structural coping methods, such as home insulation and availability of air conditioning, may have for aging adults who are physiologically more susceptible to effects of extreme temperature.

The physiological effects of exposure to cold can be modified by many factors including climate, season, and housing conditions in the area [5, 49]. Our findings suggest there is unlikely to be a universally optimal temperature for cognitive performance, but rather the temperature relative to the area’s normative climate is crucial. Cold exposure while performing a task, in particular for those not accustomed to such temperatures, can result in discomfort and an inability to concentrate. This distraction hypothesis is evidenced in previous studies by higher reported rates of discomfort when exposed to colder temperatures [38]. More recent work suggests that acute cold exposure may reduce levels of catecholamines or thyroid hormones, which in turn is associated with worse cognitive functioning [50, 51].

Unlike previous studies, we did not find a relationship between higher temperatures and cognitive function. As mentioned above, Dai and colleagues found both cold and heat effects of temperature on cognition in 594 older men from 2000 to 2008 [17]. One explanation may be that our cognitive measures were gathered more recently, and effective air conditioning prevalence (i.e., protection from outdoor high temperatures) has increased over time. In future research, we aim to gather information necessary to assess effect modification of the temperature-cognition association by air conditioning as well as heater/furnace ownership.

In the adjusted models, we observed that late summer and fall were associated with better cognitive performance. Our findings are consistent with the limited prior research on seasonality and cognition. In a study of ~ 3000 older adults from Canada, the United States and France, participants evaluated in the winter/spring (January–June) had higher odds of meeting the clinical criteria for dementia or mild cognitive impairment than those examined in the summer or fall [15]. A previous cross-sectional study in older adults from the REGARDS cohort also noted a significant adverse effect of spring and winter on cognition [13].

This study used a large national sample of aging adults, a previously understudied and vulnerable population in the context of temperature and cognitive performance. We used temperature data obtained directly from weather stations, which reflects temperature exposure for an individual. We accounted for regional confounding with regionally varying individual-level covariates and examined effect modification by region. We modeled the primary exposure measure using flexible statistical techniques. Thus, we were able to more accurately describe the temperature-cognition relationship, which may not be captured by categorical or linear functional forms. Additionally, we accounted for the temporal effect of the exposure–response relationship, allowing us to examine the cognitive effects of temperatures preceding the day of testing. Our study was cross-sectional in nature. However, some common concerns regarding temporality that often accompany this study design were mitigated in this study. First, our outcome of cognition is unlikely to influence our primary exposure of outdoor temperatures. Furthermore, we found that lag days beyond lag day 2 were not significant; thereby suggesting that the temperature exposure that is associated with cognitive change is a short-term temperature exposure that precedes the cognitive measurement. As a result, this strengthens the ability to draw conclusions from the findings of this study, in spite of the cross-sectional nature. Lastly, unlike previous studies, we were able to account for the effects of season and temperature simultaneously.

Our study should be interpreted in the context of some limitations. First, participants completed cognitive assessments indoors where conditions may be climate-controlled. Using outdoor temperature for our exposure measure may have resulted in some exposure misclassification. However, prior research, though limited geographically, has shown that outdoor temperatures correlate well with indoor temperatures in at least some locations in the U.S., such as Massachusetts (e.g., Nguyen and colleagues (2014) [52]). Second, we lacked data on sunlight, which previous literature has found to be associated with cognitive performance [13]. We did include seasonality within our regression model, and other studies in older adults in the REGARDS cohort have shown that season and sunlight are well correlated [13]. Nonetheless, residual confounding cannot be ruled out. Third, we were unable to account for the role of air conditioning or heating availability in households. There does not currently exist a national database of air conditioner use, and data on the household characteristics of REGARDS participants is not currently available. These may represent important temperature-related coping mechanisms for aging adults and have been identified as important effect modifiers in previous work [16, 53]. In the current work, we aimed to examine associations between temperature and cognition, accounting for potential confounding by age, race, sex, educational attainment (a measure of socioeconomic status), and climate zone as well as effect modification by climate zone. Effect modifiers (e.g., poverty and housing), and whether housing characteristics mediate the association between poverty and cognition were outside the scope of this current study. They represent distinct, but important research questions, given our findings in this study. In future work, we hope to further study this research question by linking to housing data in order to derive air conditioning availability using a new model of air conditioning availability or rely on participant self-reported data in order to incorporate this information [54].

## Conclusion

We conducted the first observational study in a national cohort of aging adults examining the association between exposure to outdoor temperatures and cognitive functioning across regions. Our novel findings of adverse cognitive associations between cold temperature exposure and potential beneficial relationship between late summer/early fall seasons, and cognition should be further examined in longitudinal studies. While some of the observed estimates were modest in magnitude, the results still have potential implications for the practices of public health, policymakers and clinicians. Future weather preparedness and management plans for events such as cold snaps or polar vortexes, in particular in traditionally milder regions of the country, may consider incorporating and emphasizing cold weather and season on the cognitive health of aging adults. At least for short-term extreme cold events, this can have implications for the design of educational programs and include outreach to older adults and their caregivers to prepare for such events. Findings from future studies can help to further inform the need and/or design for such policies and programs. Furthermore, clinical diagnosis, support, and care for cognitively impaired older adults may need to vary by time of year (season) and weather conditions. It is possible that this need would be greatest for older adults without access to comfortable indoor conditions. Further research is necessary to elucidate the mechanisms driving the observed associations and understanding the environmental risk factors and social, housing, and built environment modifiers. Insight into the mechanism can help inform policy decisions and interventions to mitigate adverse cognitive impacts of exposure to short-term changes in temperature.

## Supplementary Information

**Additional file 1: **Contains 3 additional figures that support decisions made in the analysis. **Figure 1.0.** Association between temperature on day of testing and composite cognitive score with temperature modeled using natural cubic splines with 2 (A), 3 (B), 4 (C), 5 (D), 6 (E), and 7 (F) degrees of freedom. **Figure 2.0.** Association between temperature and composite cognitive score. Temperature value on the one (A), two (B), three (C), four (D), five (E), six (F), and seven (G) lag days were considered. **Figure 3.0.** Association between temperature on day of testing (A), cumulative effect of temperature on day of testing and 1 day prior (B) and cumulative effect of temperature on day of testing and 2 days prior (C), and composite cognitive score.

## Data Availability

REGARDS data that support the findings of this study are available from the principal investigators of REGARDS at the University of Alabama at Birmingham but restrictions apply to the availability of these data, which were used under license for the current study, and so are not publicly available. Applications can be submitted to the REGARDS executive committee for access to the REGARDS data. The weather station data is available on the National Neighborhood Data Archive (NaNDA): https://www.openicpsr.org/openicpsr/nanda.

## Abbreviations

CI: Confidence Intervals
DF: Degrees of Freedom
DLNM: Distributed Lag Non-linear Models
MD: Mean Difference
MMSE: Mini-Mental State Exam
WLD: Word List Delayed Recall
WLL: Word List Learning
REGARDS: REasons for Geographic and Racial Differences in Stroke
US: United States of America
