# Supplementary material for: Association between temperature exposure and cognition: a cross-sectional analysis of 20,687 aging adults in the United States
Source: BMC Public Health. 2021 Jul 29;21:1484. doi: 10.1186/s12889-021-11533-x (PMC8323228; doi:10.1186/s12889-021-11533-x)
Supplement: Supplementary file 1 — Additional file 1: Contains 3 additional figures that support decisions made in the analysis. Figure 1.0. Association between temperature on day of testing and composite cognitive score with temperature modeled using natural cubic splines with 2 (A), 3 (B), 4 (C), 5 (D), 6 (E), and 7 (F) degrees of freedom. Figure 2.0. Association between temperature and composite cognitive score. Temperature value on the one (A), two (B), three (C), four (D), five (E), six (F), and seven (G) lag days were considered. Figure 3.0. Association between temperature on day of testing (A), cumulative effect of temperature on day of testing and 1 day prior (B) and cumulative effect of temperature on day of testing and 2 days prior (C), and composite cognitive score. [file 12889_2021_11533_MOESM1_ESM.docx]

**DATA SUPPLEMENT**

**Association Between Temperature Exposure and Cognition: A Cross-sectional Analysis of 20,687 Aging Adults in the United States**

**Contents:**

**Figure 1** Association between temperature and cognition with temperature modeled using between 2-7 degrees of freedom.

**Figure 2** Association between temperature and composite cognitive score, considering temperature value on the day of testing and the 1-7 days before day of testing (lag days).

**Figure 3** Association between temperature on day of testing, cumulative effect of temperature on day of testing and one day prior and cumulative effect of temperature on day of testing and two days prior, and composite cognitive score.

**
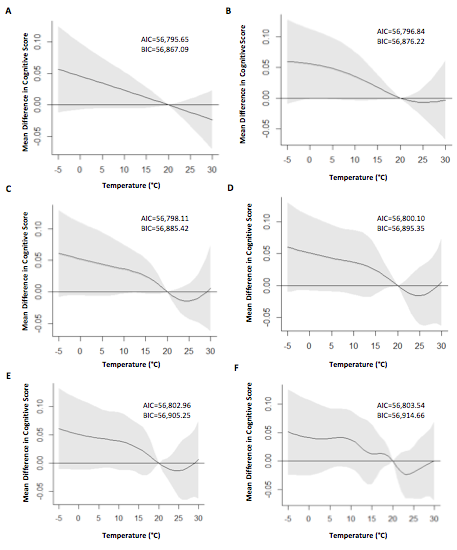
**

**Figure 1.0** Association between temperature on day of testing and composite cognitive score with temperature modeled using natural cubic splines with 2 (A), 3 (B), 4 (C), 5 (D), 6 (E), and 7 (F) degrees of freedom. Models were adjusted for season and climate region. Grey shaded area represents the 95% confidence interval around the effect estimate. AIC, Akaike Information Criterion; BIC, Bayesian Information Criterion


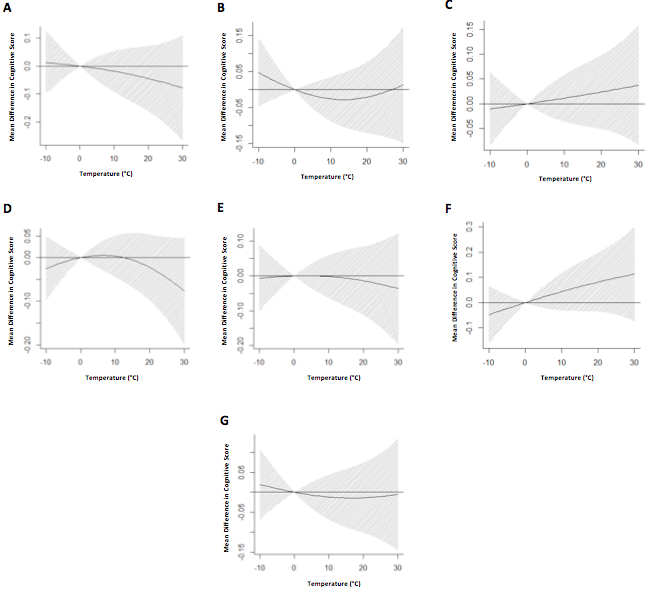


**Figure 2.0** Association between temperature and composite cognitive score. Temperature value on the one (A), two (B), three (C), four (D), five (E), six (F), and seven (G) lag days were considered. Models were adjusted for season, age at time of test, education level, region, race and gender. Temperature was modeled using 2 degrees of freedom. Grey shaded area represents the 95% confidence around the effect estimate.


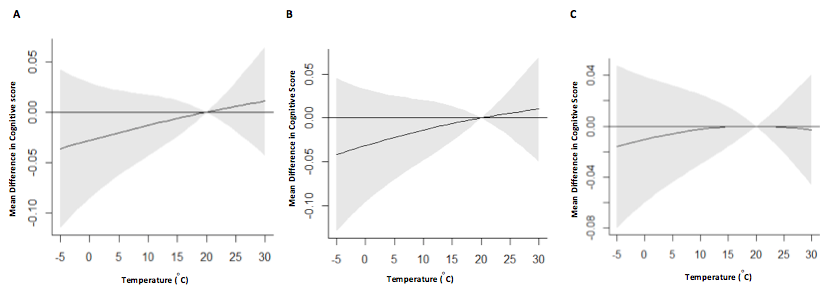


**Figure 3.0** Association between temperature on day of testing (A), cumulative effect of temperature on day of testing and one day prior (B) and cumulative effect of temperature on day of testing and two days prior (C), and composite cognitive score. Reference value for temperature was 20°C. Models adjusted for age at time of cognitive assessment, year of test, season, education status at baseline, sex, race and climate region. Grey shaded area represents the 95% confidence around the effect estimate.
